# Supplementary figures and images for: Geometric-Phase Microscopy for Quantitative Phase Imaging of Isotropic, Birefringent and Space-Variant Polarization Samples
Source: Sci Rep. 2019 Mar 5;9:3608. doi: 10.1038/s41598-019-40441-9 (PMC6401004; doi:10.1038/s41598-019-40441-9)

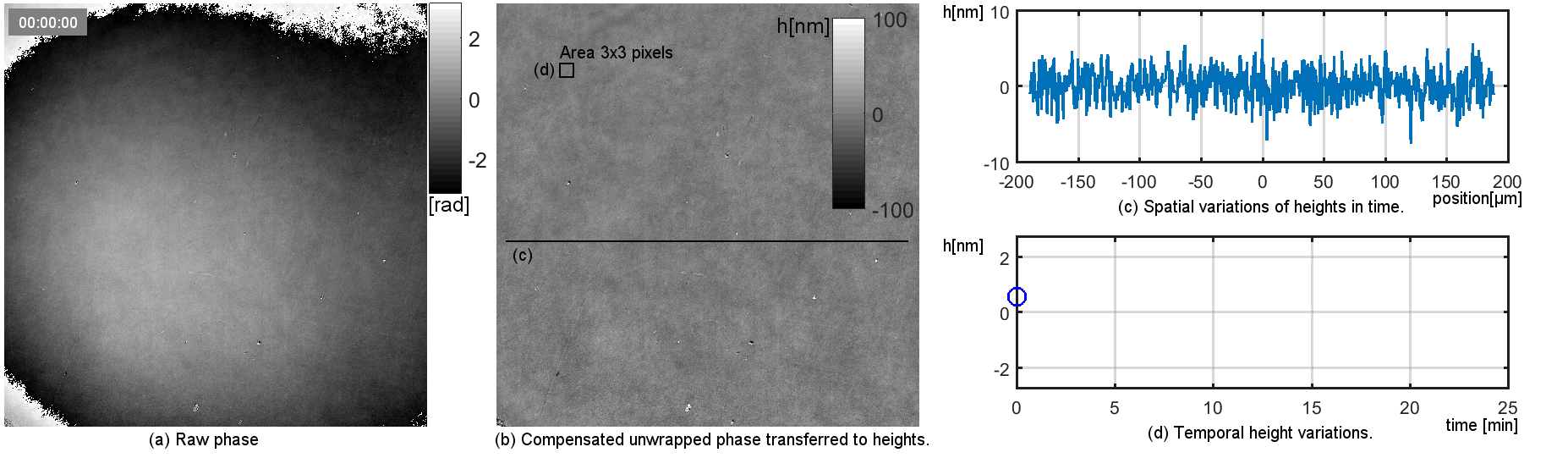

Supplement: Supplementary file 1 — Video 1 [file 41598_2019_40441_MOESM1_ESM.gif]

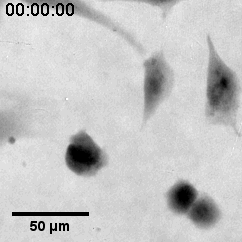

Supplement: Supplementary file 2 — Video 2 [file 41598_2019_40441_MOESM2_ESM.gif]

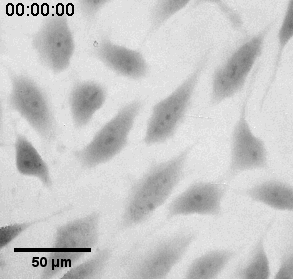

Supplement: Supplementary file 3 — Video 3 [file 41598_2019_40441_MOESM3_ESM.gif]

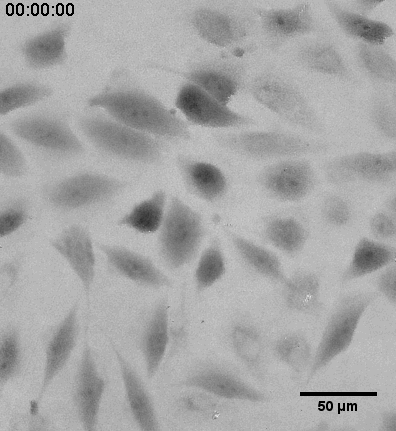

Supplement: Supplementary file 4 — Video 4 [file 41598_2019_40441_MOESM4_ESM.gif]
