# Supplementary material for: Geometric-Phase Microscopy for Quantitative Phase Imaging of Isotropic, Birefringent and Space-Variant Polarization Samples
Source: Sci Rep. 2019 Mar 5;9:3608. doi: 10.1038/s41598-019-40441-9 (PMC6401004; doi:10.1038/s41598-019-40441-9)
Supplement: Supplementary file 5 — Supplementary information [file 41598_2019_40441_MOESM5_ESM.pdf]

Supplementary information

Geometric-phase microscopy for quantitative phase imaging of isotropic, birefringent and space-variant polarization samples

Petr Bouchal,[1,2,*] Lenka Štrbková,[2] Zbyněk Dostál,[1,2] Radim Chmelík[1,2] & Zdeněk Bouchal[3]

[1]Institute of Physical Engineering, Faculty of Mechanical Engineering, Brno University of Technology, Technická 2, 616 69 Brno, Czech Republic
[2]Central European Institute of Technology, Brno University of Technology, Purkyňova 656/123, 612 00 Brno, Czech Republic
[3]Department of Optics, Palacký University, 17. listopadu 1192/12, 771 46 Olomouc, Czech Republic
*petr.bouchal@vutbr.ceitec.cz

Part A
Modification of optical microscope for Q4GOM live cell imaging

Quantitative 4G optics microscopy (Q4GOM) allows to quantify the retardance of sample and reference waves having orthogonal polarizations and entering the add-on 4G optics imaging module. In experiments with polarization sensitive samples, the 4G optics module is connected to a standard optical microscope. When imaging isotropic samples, the 4G optics module is connected to a microscope working with the polarization adapted interference Mirau microscope objective (MMO). In Figure S1, the modified microscope is shown that works with the polarization MMO and has been used in demonstrated live cell experiments.

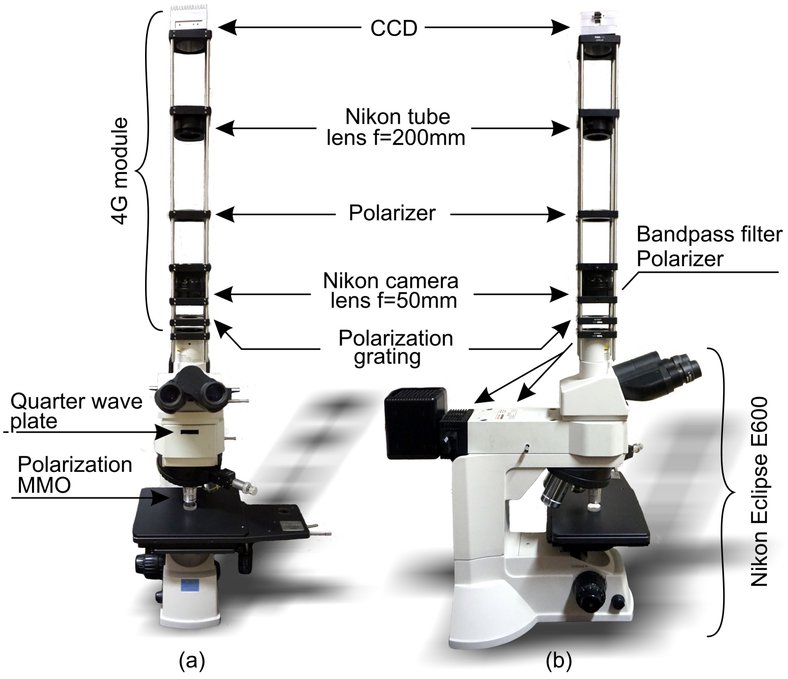

Figure S1. Photographs of the adapted Nikon Eclipse L150 microscope operating in the polarization-division multiplexing mode used in Q4GOM: (a) front view, (b) side view.

The setup is based on the standard microscope Nikon Eclipse L150 working with the episcopic illumination. The polarization adaptation of the microscope involves removing the CCD and connecting the 4G optics imaging module to its original position. The adjustment of the microscope and the 4G optics module is performed simultaneously by exactly placing the geometric-phase grating (GPG) at the plane, where the sharp image of the sample is created by the MMO and the tube lens. The polarization adapted MMO is standardly attached at the objective turret. The quantitative phase measurement is easily initiated by focusing the specimen without any further adjustment procedures needed when double-path off-axis techniques are applied.

The 4G optics module is composed of the cage system Thorlabs, the custom-made polarization GPG (Boulder Nonlinear Systems) and two Nikon lenses forming a 4f system. The steering angle between +1st and -1st diffraction order of the GPG and the magnification of the 4f system are optimally chosen to create the carrier frequency enabling spatial separation of the interfering waves in the Fourier domain. In the final design, the steering angle of 8.16° was chosen and combined with the magnification 4x of the 4f system. To maintain the proper carrier frequency in the entire field of view, the 4G optics module was designed to minimize optical aberrations. Hence, well corrected Nikon camera objective (f=50 mm) and Nikon tube lens (f=200 mm) were used as the first and second lenses of the 4f system.

Part B
Polarization modification of interference Mirau microscope objective

In the quantitative phase imaging (QPI) of isotropic samples, the polarization coding of the sample and reference waves was carried out using the interference MMO (Nikon, 10x, NA=0.3), which was modified by the polarization components. The aim of the polarization adaptation is to create the output signal and reference waves with the orthogonal circular polarizations from a linearly polarized illumination wave. This is achieved by placing the quarter wave plates QWP1 and QWP2 above and below the beam splitter BS1 and setting their fast axes in suitable directions, as shown in Figure S2. The fast axis of the QWP1 coincides with the polarization direction of the incident linearly polarized wave; hence the polarization state of the reference wave created by the reflection on the reference mirror M and the double reflection on the BS1 maintains the initial linear polarization. Because the fast axis of the QWP2 is rotated by 45°, the signal wave created by the double passing through the QWP2 and the reflection at the sample acquires linear polarization orthogonal to the input linear polarization. Using additional QWP3 outside the objective, the orthogonal linear polarizations can be transformed into orthogonal circular polarizations.

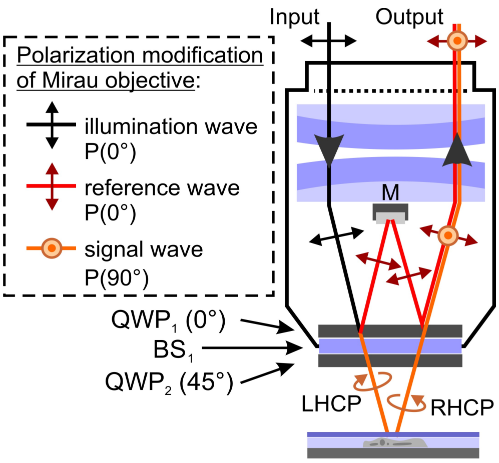

Figure S2. Creation of sample and reference waves with orthogonal linear polarizations by the polarization adapted Mirau interference microscope objective.

To switch the operation of the MMO to a polarization mode, the adapter consisting of the QWP1, QWP2 and BS1 was designed and manufactured. Using the rotating rings with the locking screws, the angular orientation of the quarter wave plates can be adjusted with respect to the input polarization and locked at the optimal position. The compact adapter is movable towards the reference mirror M by means of an external thread. This shift allows adjusting the optical path difference between the signal and reference waves. The 3D model of the polarization adapter assembled with the MMO is shown in Figure S3a together with the description of the individual components. Photograph of the polarization adapted MMO is in Figure S3b.

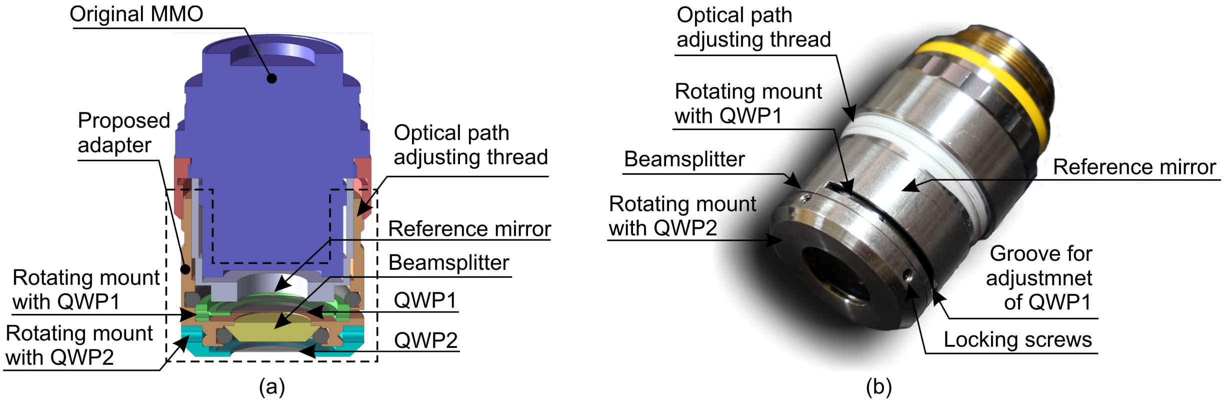

Figure S3. Polarization adapted Mirau interference microscope objective. (a) 3D model of thecompact polarization adapter. (b) Photograph of Mirau objective with the mounted polarization adapter.

Part C
Calculation model for the phase retardance restoration

To optimize the experimental setup, a computational simulation model of Q4GOM was created. In this model, the operation of the 4G optics module is described including the polarization-selective transformation of light by the GPG, the achromatic recording of the off-axis correlation patterns and the restoration of the phase retardance. The computational model outlined here also describes the illumination of isotropic samples and the preparation of polarization coded waves by means of the polarization adapted MMO. This part of the simulation model is omitted in the imaging of polarization sensitive samples because the polarization MMO is not used.

A thin GPG placed in the input plane of the 4G module is illuminated by the image field $\mathbf{E} = \mathbf{E}(\mathbf{r}_\perp, t)$ created by the polarization adapted microscope using broadband spatially incoherent light. When the microscope is equipped with achromatic polarization elements, the monochromatic components of the microscope image field $\mathbf{e} = \mathbf{e}(\mathbf{r}_\perp, \nu)$ with the frequency ν are composed of the polarization coded sample and reference waves, $\mathbf{e} = U_S \mathbf{J}_\mathbf{L} + U_R \mathbf{J}_\mathbf{R}$, where $\mathbf{J}_\mathbf{L}$ and $\mathbf{J}_\mathbf{R}$ denote the Jones vectors for LHCP and RHCP, respectively. $U_S = U_S(\mathbf{r}_\perp, \nu)$ and $U_R = U_R(\mathbf{r}_\perp, \nu)$ are the complex amplitudes of the sample and reference waves obtained by the convolution $U_j = (R_j U_I) \otimes G_j$, $j = S, R$, where U_I is the monochromatic component of the illuminating field and G_j is the impulse response function determined for the sample and reference imaging paths formed by the MO and the tube lens (under ideal imaging conditions the same impulse response function G is assigned to both paths). R_S represents a spatially variable complex reflection function of the sample, while R_R is a constant reflection coefficient of the mirror M placed on the front surface of the MO. The thin GPG modulating the geometric phase of the microscope image field and transforming its polarization state can be described using Jones calculus [1,2]. This approach is valid for assumptions of large grating period, normal incidence and infinite lateral grating dimensions. The GPG then can be described as a wave plate with phase retardation $\Delta\Gamma$ and spatially varying angle of the anisotropy axis, $\varphi = \varphi(x)$. While $\Delta\Gamma$ is frequency dependent, $\Delta\Gamma = \Delta\Gamma_0 \nu / \nu_0$, the spatial modulation of φ is achromatic, $\varphi = 2\pi x / \Lambda$. Here $\Delta\Gamma_0 = \pi$ is the phase retardation introduced between linear polarization components for the central frequency ν_0 of the spectrum, Λ is the period of the linear spatial change of the angle of the anisotropy axis, and x is the Cartesian coordinate at the plane of the GPG. The spatially varying Jones matrix describing operation of the GPG in the linear polarization basis is given as $W_{LP} = R_{-\varphi} W_0 R_\varphi$, where R_φ and $R_{-\varphi}$ are the rotation matrices and W_0 is the matrix of a wave plate with the phase retardation $\Delta\Gamma$ and the anisotropy axis oriented in the angle $\varphi = 0$. Since the microscope field is composed of waves with the LHCP and RHCP, the matrix $W_{CP} = T^{-1} W_{LP} T$ is assigned to the GPG, where the matrix T and the inverse matrix T^{-1} provide transition to the circular polarization basis. The microscope image field modulated by the GPG and evaluated just behind the grating is given by the matrix transformation $\mathbf{e}_\mathbf{G} = W_{CP} \mathbf{e}$, and can be written as

$$\mathbf{e_G} = \left[U_S^{(0)} + U_R^{(1)} \exp\left(-i\frac{4\pi}{\Lambda} x \right) \right] \mathbf{J_L} + \left[U_R^{(0)} + U_S^{(-1)} \exp\left(i\frac{4\pi}{\Lambda} x \right) \right] \mathbf{J_R},$$

where

$$U_S^{(0)} = \eta_0^{1/2} U_S,\ U_R^{(0)} = \eta_0^{1/2} U_R,\ U_R^{(1)} = KU_R,\ U_S^{(-1)} = KU_S,$$

and $K = e^{-i\frac{\pi}{2}} \left(\eta^{(1)} + \eta^{(-1)} \right)^{1/2}$.A portion of the energy of the signal and reference waves, which is determined by the zero-order diffraction efficiency $\eta^{(0)}$, remains unaffected both in the polarization state and the propagation direction. The signal and reference waves diffracted by the GPG interchange their polarization states, hence the LHCP of the signal wave turns to the RHCP and vice versa.The total amount of light energy that diffracts to $+1$ st and -1 st diffraction order is determined by $\eta^{(1)} + \eta^{(-1)} = 1 - \eta^{(0)}$, where $\eta^{(0)} = \cos^2\left(\pi\nu / 2\nu_0 \right)$. For monochromatic illumination with the central frequency ν_0, the undiffracted light disappears and all energy is directed to the $+1$ st and -1 st diffraction orders. For the spectral range of 50 nm used in Q4GOM, the efficiency $\eta^{(1)} + \eta^{(-1)}$ safely reaches 99.5%. The zero-order light is insignificant in this case and does not impair the imaging. The spatial light modulation introduced by the GPG during the polarization transformation results in the angular separation of the signal and reference waves that is apparent in the far field. In the 4G module, where a Fourier lens L_1 is used, the separation of waves can be demonstrated in the field $\boldsymbol{\varepsilon}_\mathbf{G} = \mathrm{FT}_1\left\{ \mathbf{e_G} \right\}$ available at the back focal plane of the lens L_1. The field $\boldsymbol{\varepsilon}_\mathbf{G}$ represents the spatial spectrum of $\mathbf{e_G}$, and FT_1 denotes the Fourier transform realized by the lens L_1. Neglecting low zero-order light, $\boldsymbol{\varepsilon}_\mathbf{G}$ can be written by $\boldsymbol{\varepsilon}_\mathbf{G} = \varepsilon_G^{(1)} \mathbf{J_L} + \varepsilon_G^{(-1)} \mathbf{J_R}$, where

$$\varepsilon_G^{(1)} = Ku_R\left(x_1 - \frac{2\lambda f_1}{\Lambda} \right),\ \varepsilon_G^{(-1)} = Ku_S\left(x_1 + \frac{2\lambda f_1}{\Lambda} \right).$$

The spatial spectra $u_R = \mathrm{FT}_1\left\{ U_R \right\}$ and $u_S = \mathrm{FT}_1\left\{ U_S \right\}$ are shifted along the x_1 axis of the back focal plane of the lens L_1 in opposite directions. The shift is spectrally dependent, and its magnitude is proportional to the wavelength $\lambda = c/\nu$ (c being speed of light in vacuum) and the focal length f_1. Prior to detection of the image field, the Fourier transform by the lens L_2 with the focal length f_2 is performed, followed by the polarization transformation using a linear polarizer. The Fourier transformed field is given by $\mathbf{e}' = \mathrm{e}'^{(1)} \mathbf{J_L} + \mathrm{e}'^{(-1)} \mathbf{J_R}$, where

$$\mathrm{e}'^{(1)} = KU_R \exp\left(i\frac{4\pi}{\Lambda'} x' \right),\ \mathrm{e}'^{(-1)} = KU_S \exp\left(-i\frac{4\pi}{\Lambda'} x' \right).$$

The phase modulation in $+1$ st and -1 st diffraction order is achromatic and the spatial period is given by $\Lambda' = m\Lambda$, where $m = f_2 / f_1$ denotes the magnification of the lenses L_1 and L_2. After polarization projection to the angle ϑ, the resulting image field $\mathbf{E}' = \mathbf{E}'\left(\mathbf{r}'_\perp, t \right)$ is created in the output plane of the 4G module

$$\mathbf{E}' = \left[\mathrm{E}'_R \exp\left(i\frac{4\pi}{\Lambda'} x' + i\vartheta \right) + \mathrm{E}'_S \exp\left(-i\frac{4\pi}{\Lambda'} x' - i\vartheta \right) \right] \mathbf{J}_\vartheta,$$

where $\mathbf{J}_\vartheta = \left[\cos(\vartheta)\ \ \sin(\vartheta) \right]^T$ and $\mathrm{E}'_j = \mathrm{E}'_j\left(\mathbf{r}'_\perp, t \right),\ j = S, R,$ are complex amplitudes of the signal and reference waves obtained by the Fourier transform of the temporal-frequency spectra, $\mathrm{E}'_j = \left(1/\sqrt{2} \right) \mathrm{FT}\left\{ KU_j \right\},\ j = S, R.$ The detected intensity can then be written as

$$I' = I'_S + I'_R + \Gamma_{S,R}(\tau) \exp\left(-i\frac{8\pi}{\Lambda'} x' - i2\vartheta \right) + \Gamma^*_{S,R}(\tau) \exp\left(i\frac{8\pi}{\Lambda'} x' + i2\vartheta \right),$$

where $I'_j = \left\langle \left| \mathrm{E}'_j \right|^2 \right\rangle,\ j = S, R$, denotes the intensity of the sample and reference field, respectively, and $\Gamma_{S,R}(\tau) = \left\langle \mathrm{E}'_S(t) \mathrm{E}'^*_R(t + \tau) \right\rangle$ represents the space-time mutual coherence function describing the spatial and temporal correlation between these fields. The entire intensity I' is an off-axis correlation pattern recorded with the frequency independent carrier, hence $\Gamma_{S,R}$ can be separated using the Fourier filtering. The phase $\Delta\Phi$ directly connected to the ground-truth variations of the optical path length caused by light interaction with the sample is

determined as $\Delta\Phi = \arg\{\Gamma_{S,R}(0)\}$. Because a pure polarization coded reference wave is used, the phase reconstruction is completely decoupled from amplitude variations caused by the sample. By this way, the highly-accurate restoration of the ground-truth phase retardance limited only by diffraction effects and coherence properties of light is achieved. This is clearly documented provided the monochromatic spatially incoherent light and ideal optical elements with infinite aperture are assumed to be used. The monochromatic illuminating field is specified by the delta function as $U_I(\mathbf{r}_{\perp 0},\nu) = \delta(\nu - \nu_0)U_I(\mathbf{r}_{\perp 0})$, where $\mathbf{r}_{\perp 0} = (x_0, y_0)$ is vector of the lateral coordinates at the sample plane. When the light is spatially incoherent, only optically conjugate points of the sample and reference plane are correlated, hence $\langle U_I(\mathbf{r}_{\perp 0})U_I^*(\mathbf{r}'_{\perp 0})\rangle = \delta(\mathbf{r}_{\perp 0} - \mathbf{r}'_{\perp 0})I_I(\mathbf{r}_{\perp 0})$, where I_I is the illumination intensity. In this case, the mutual coherence function can be written as

$$\Gamma_{S,R}(0) = \int_{-\infty}^{+\infty}\int_{-\infty}^{+\infty} I_I(\mathbf{r}_{\perp 0})R_S(\mathbf{r}_{\perp 0})R_R^* \left|G(\mathbf{r}'_{\perp} - M\mathbf{r}_{\perp 0})\right|^2 d\mathbf{r}_{\perp 0},$$

where M is the magnification of the entire system, R_S and R_R denote a complex reflection function of the sample and a constant reflection coefficient of the reference mirror, respectively, and G is the impulse response function of the entire system including both the microscope and the 4G module (under ideal imaging conditions G is the same for sample and reference paths). In an ideal system with infinite aperture, the point imaging is achieved and G becomes the delta function, hence $\Gamma_{S,R}(0) = I_I(\mathbf{r}'_{\perp}/M)R_S(\mathbf{r}'_{\perp}/M)R_R^*$. Up to a constant given by the reflection coefficient of the reference mirror, the restored phase accurately maps the changes in the optical path caused by the sample, $\Delta\Phi \propto \arg\{R_S\}$.

Part D
Reconstruction of the holograms

Thanks to the 4G optics module, Q4GOM provides the instantaneous quantitative phase imaging. The imaging performance is based on the common-path off-axis holography working with the achromatic carrier frequency. The quantitative images are reconstructed from a single hologram using procedure proposed by Zikmund et al. [3] and schematically illustrated in Figure S4.

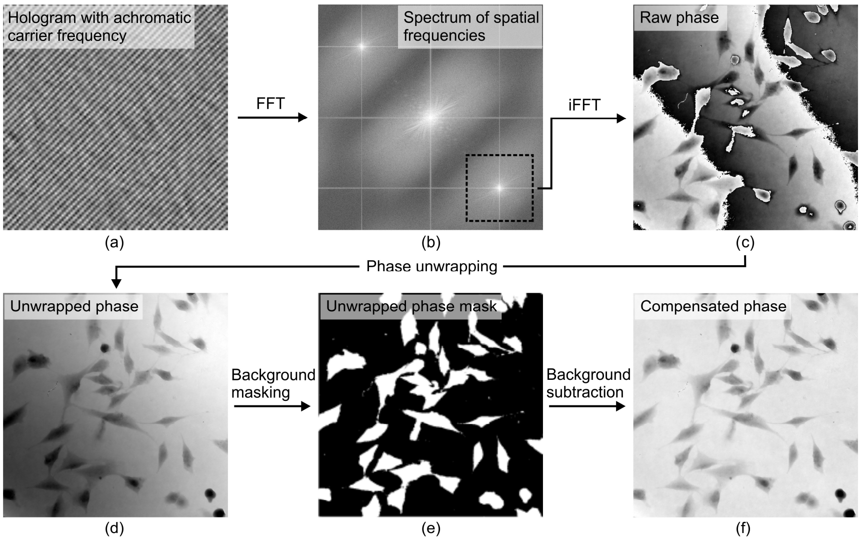

Figure S4. Principle of hologram reconstruction in Q4GOM. (a) Enlarged part of the hologram with achromatic carrier frequency. (b) Spectrum of spatial frequencies obtained by Fourier transform of the recorded hologram. (c) Raw phase reconstructed by inverse Fourier transform of the area around the carrier frequency. (d) Unwrapped phase image and (e) unwrapped phase mask obtained from raw phase. (f) Resulting quantitative phase image with compensated background phase.

Hologram with the achromatic carrier frequency (Figure S4a) is Fourier transformed and the spatial spectrum is obtained (Figure S4b). The spatial spectrum of the true image that is separated by the carrier frequency is cut out (square box in Figure S4b) and after application of inverse Fourier transform the raw phase image is reconstructed

(Figure S4c). The raw phase is unwrapped using Goldstein unwrapping method (Figure S4d). The unwrapped phase image is segmented and regions corresponding to the background are detected and masked (Figure S4e). The phase values from the background regions are processed by the polynomial surface fitting via the least-square method and subtracted from the unwrapped phase image. The presented results were obtained with 3rd order polynomial. In the time lapse imaging, the polynomial fitting and background phase compensation are realized in the frame to frame basis. The whole procedure provides compensated quantitative phase image (Figure S4f). Although the common-path Q4GOM has a weak disturbing background compared to double-path systems, the phase compensation algorithm eliminates minor wavefront distortions and thus allows direct evaluation of the true phase.

The linear phase mismatch apparent in Figure S4c was intentionally introduced during the hologram reconstruction by a slight shift of the window function in Figure S4b with regard to the carrier frequency. This was done to demonstrate graphically the principle and benefits of the background compensation [apparent difference between Figure S4(d) and Figure S4(f)]. The true residual distortion of the background phase is demonstrated in Supplementary video 1.

Part E
Evaluation of the precision and accuracy

Q4GOM offers a unique option to create the off-axis correlation records in a common-path arrangement using a pure reference wave; hence the ground-truth optical path difference can be reconstructed from a single-shot.

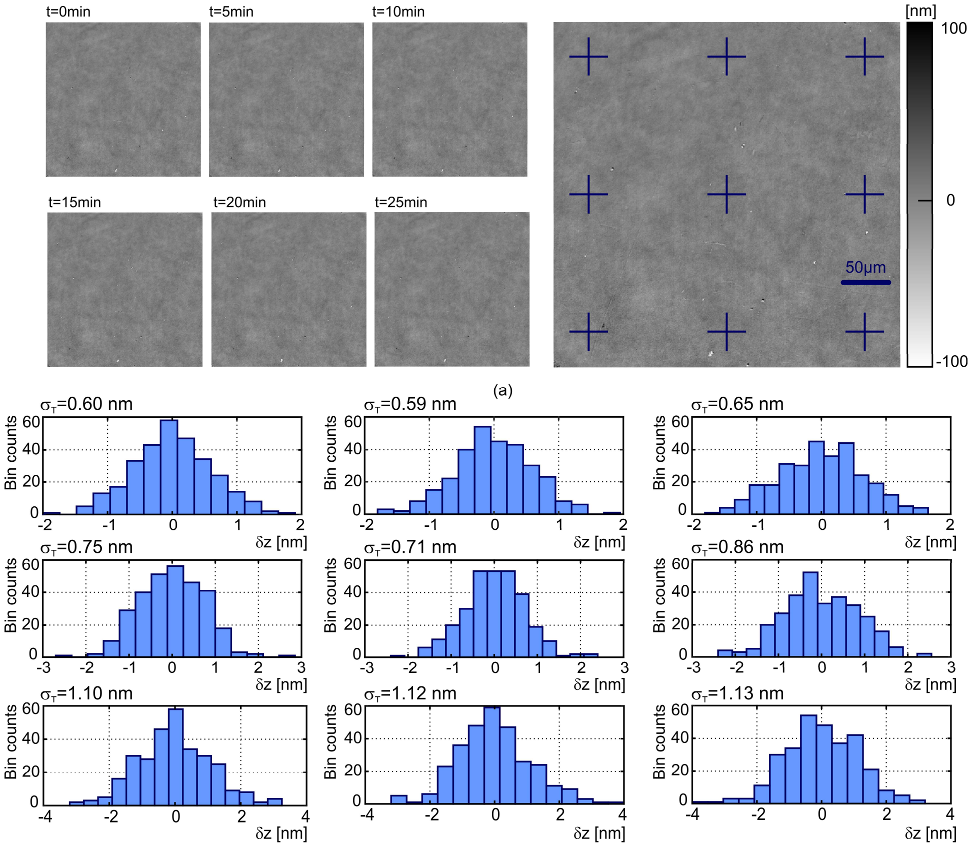

Figure S5: Evaluation of the temporal stability with automated compensation of the background phase. (a) Representative unwrapped phase images with compensated background phase. Images were taken from the series of 300 records captured during 25-minute-long time period. Enlarged image with "+" marks shows areas, where the temporal stability of the compensated unwrapped phase was evaluated. (b) Histograms demonstratingtemporal variations of the reconstructed height.

Thanks to the exceptional robustness of the setup and the use of the pure reference wave, the optical performance achieved in Q4GOM meets the highest demands on the precision and accuracy of the phase measurement. To demonstrate the performance of the phase imaging, we carried out experiments demonstrating the temporal stability and the spatial background noise of the restored phase. In Figure S5 and Figure S6, the experimental results supporting analysis presented in the main text are demonstrated.

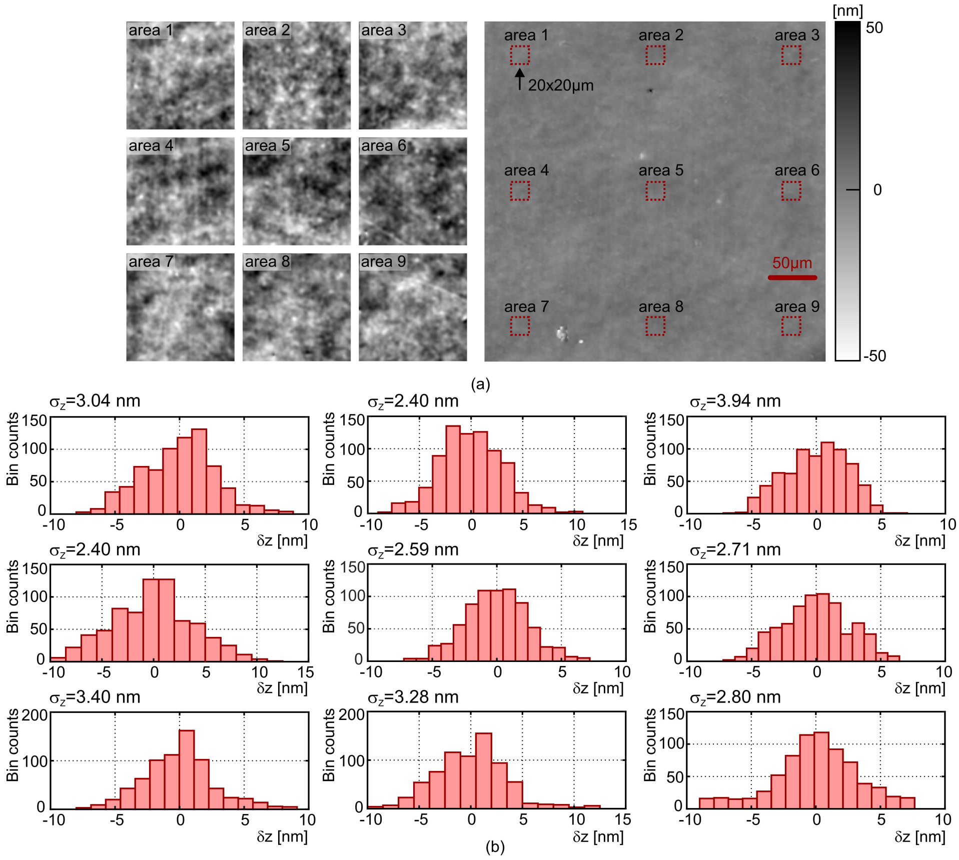

Figure S6: Evaluation of the spatial background noise. (a) Representative unwrapped phase image with the compensated background phase and enlarged images of the areas 1 to 9. The enlarged areas were min-max scaled. (b) Histograms of the height variations at the examined areas.

Part F
Preparation of biological specimens

In the demonstration and advanced experiments presented in the main text, we used different types of cells including human cheek cells, human red blood cells and spontaneously transformed rat embryonic fibroblast (LW3K12) cells. The human cheek cells were scraped from mouth, placed on the plane mirror and used for preparation of a standard wet mount. The specimen with red blood was obtained smearing the drop of blood over the plane mirror slide instead of the microscope glass slide. The LW13K2 cells were firstly grown attached to a solid surface and maintained in Eagle's minimal essential medium (Sigma-Aldrich, Czech Republic) supplemented with 10% fetal bovine serum (Sigma-Aldrich, Czech Republic) and gentamicin (Sigma-Aldrich, Czech Republic) in an incubator at 37°C and 3.5% CO_2 atmosphere. The cells were harvested by trypsinization and transferred into four mirror slides with the culture medium. The seeding densities were 80 cells/mm^2 for the first and 25 cells/mm^2 for the other three mirror slides in order to achieve confluent and sparse coverage of cells, respectively. The slides were kept in the incubator under the same conditions and imaged the following day. The first and the second slide were observed without any further intervention (experiment in Figure 3 and Figure 4 in the main text). In the third slide, culture medium was washed twice and replaced by PBS (NaCl 8 g/l, KCl 0.2 g/l, KH_2PO_4 0.24 g/l, Na_2HPO_4 1.44 g/l, pH 7.4, Sigma-Aldrich, Czech Republic). PBS deprives cells of nutrients and causes gradual morphology changes (experiment in Figure S8). In the fourth slide, culture medium was replaced by 0.25% trypsin (Sigma-Aldrich, Czech Republic), which induces rapid changes in morphology, loosening and rounding up of adhered cells (experiment in Figure S9). The cells were imaged immediately after PBS and trypsin application.

Part G
Comparison of Q4GOM cell imaging with traditional methods

To further demonstrate artifact-free image restoration and compare Q4GOM imaging with the most frequently used label-free techniques, we captured images of human cheek cells also in the Zernike phase contrast and the

differential interference contrast (DIC), respectively. The Q4GOM image of cheek cells, the enlarged image of the selected cell and the cross-section profile across the selected cell are shown in Figure S7a,b. Images of cheek cells obtained in the Zernike phase contrast and the DIC are shown in Figure S7c,d and Figure S7e,f. Zernike phase contrast images possess halo artifacts evident in both full field of view and its enlarged part, while in DIC images typical relief shading is present. Image artifacts demonstrated in Figure S7c,d and Figure S7e,f complicate automated evaluation of measured data, which is crucial for contemporary applications working with large dataset. Since the automated image evaluation is mostly based on the image segmentation, the artifact-free and quantitative nature of Q4GOM images is advantageous compared to the Zernike phase contrast and DIC images that are rather qualitative and contain artifacts. Comparing the cross-section profiles in Figure S7b,d,f, it is obvious that Q4GOM image can be easily segmented using thresholding or watershed approach, while the Zernike phase contrast and DIC images require complex algorithms due to halo and shading effect at the boundaries of the cells and rapid oscillations of the intensity within the cells. The presented results show the exceptional predestination of Q4GOM for automated study of cell phenotypes including motility, morphology or proliferation.

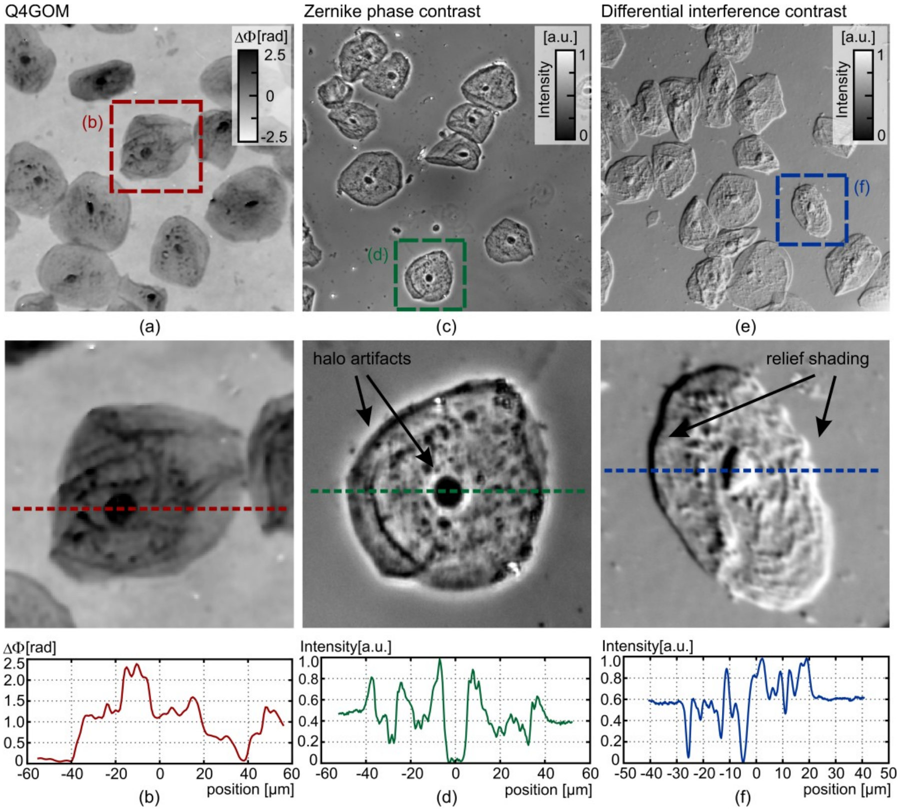

Figure S7 Comparison of the QPI in Q4GOM with the Zernike phase contrast and DIC imaging. (a) The QPI of human cheek cells. (b) Enlarged image of the cell selected from (a) and the cross-section profile along the red dashed line. (c) Zernike phase contrast image of human cheek cells. (d) Enlarged image of the cell selected from (c) showing halo artifacts at cell boundaries and around cell nucleus and the cross-section profile along the green dashed line. (e) DIC image of human cheek cells. (f) Enlarged image of the cell selected from (e) showing shading artifacts at cell and nucleus boundaries and the cross-section profile along the blue dashed line.

Part H
Advanced biological experiments

Automated cell classification

In the advanced biological experiment, LW13K2 cells were exposed to PBS that induces nutritional deprivation. The deprivation is manifested by gradual change of cell morphology. The cells were imaged by Q4GOM for 60-minute-long time period, with the time step 20 seconds. The morphological changes that occurred during the whole measurement are shown in Supplementary Movie 3. The image taken immediately after the application of PBS is shown in Figure S8a. Majority of cells in the image are viable and adhered to the surface of the mirror slide. Morphological changes of cells appeared approximately after 5 minutes after the application of PBS and most of the

cells became slightly deprived after 15 minutes, as shown in Figure S8b. Slightly deprived cells manifested shrinking while their boundaries became indented. Majority of cells became seriously deprived after 55 minutes, while obtaining a rounded morphology as demonstrated in Figure S8c. The cells were divided by the expert biologist into three classes based on their morphology: viable, semideprived and deprived cells. Figure S8d-f visualizes mentioned morphology classes of cells in each of the images in Figure S8a-c by color-coded segmentation. The color-coded images illustratively show portion of viable (orange), semideprived (green) and deprived (blue) cells at given times. The visualization was performed based on two defined morphological parameters extracted from the segmented cells: area and circularity. Figure S8g-i represents parameter plots for cells in the corresponding images in Figure S8d-f along with an exact proportion of the cells belonging to the specific classes. The straight lines dividing the three defined classes were determined by the expert biologist and remain the same for all analyzed images. The results of the experiment imply that Q4GOM offers preconditions for accurate classification of cellular morphologies which can be performed in the automated manner exploiting the methods of machine learning.

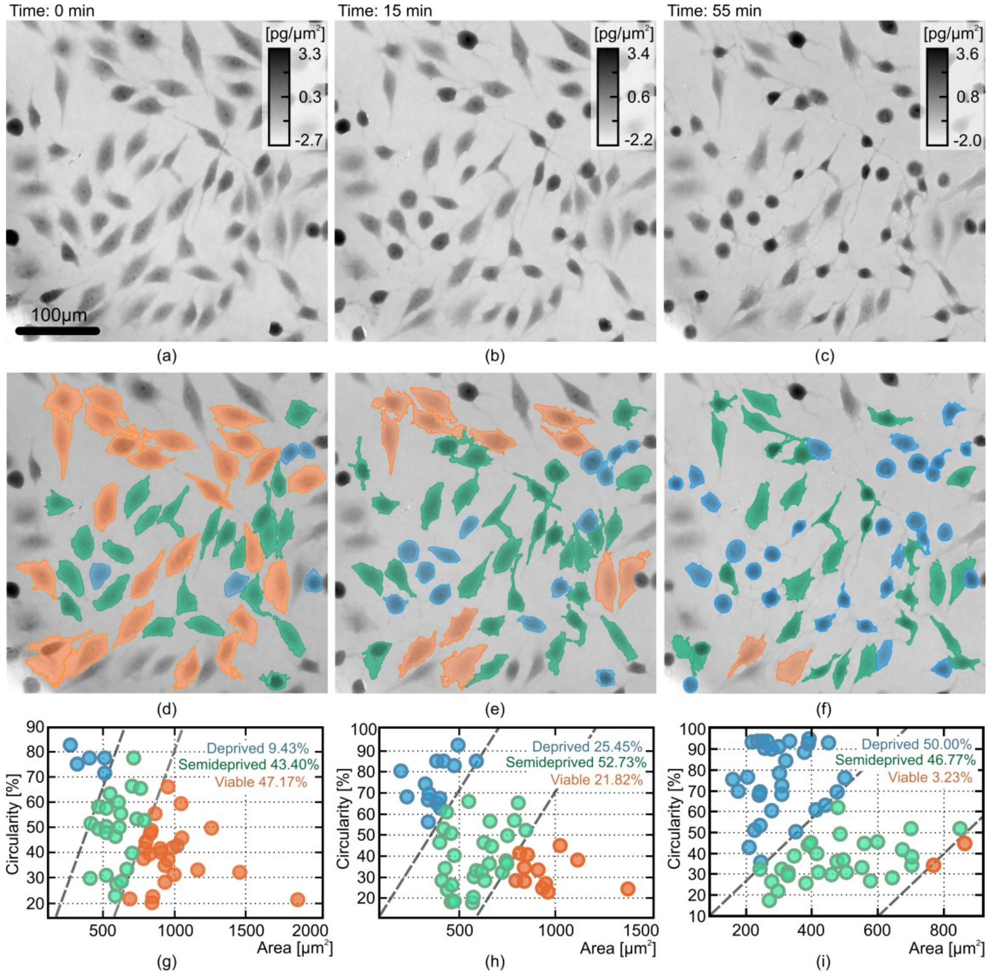

Figure S8 Morphological changes of LW3K12 cells induced by PBS. (a) Viable cells at the beginning of time-lapse imaging. (b) Semideprived cells 15 minutes after application of PBS. (c) Mostly deprived cells 55 minutes after application of PBS. (d)-(e) Segmented images of cells (a)-(c) color-coded to illustrate different cell classes (orange - viable cells, green - semideprived cells and blue - deprived cells). (g)-(i) Plots used for classification of cell morphology based on parameters of circularity and occupied area along with an exact proportion of the cells belonging to the specific classes. Images (a)-(c) are shown in units of pg/μm² recalculated from phase in radians according to Davies [4].

Visualizing cell dynamics

In the second advanced biological experiment, LW13K2 cells were exposed to trypsin that induces rapid changes in cell morphology. The cells were imaged by Q4GOM immediately after trypsin application for 1.5-minute-long time period, with the time step 1 second. The first morphology changes developed after approximately 30 seconds and were manifested mostly by the rapid shrinking and rounding up of the adherent cells and subsequent separation from

the surface of the mirror slide. These morphological changes with time step of 1 second are shown in Supplementary Movie 4. Figure S9a-d shows representative quantitative images of LW3K12 cells in four time instants separated by the time delay of 15 seconds. The relatively longer time delay was chosen to make the changes between individual images more apparent for presentation. After the segmentation of the cells from the background, the changes in cell dry mass induced by the trypsin application were visualized using dynamic phase differences method [5]. Figure S9e-g represents the color-coded differences in the cell dry mass density between the related neighboring images in Figure S9a-d (that is, Figure S9e is obtained by Figure S9a,b, etc.), while Figure S9h demonstrates cumulative changes that occurred between the states in Figure S9a and Figure S9d. The red and green colors indicate areas where the dry mass density has increased and decreased, respectively. To demonstrate changes in the dry mass density in more detail, the enlarged images of areas marked in Figure S9a-d and Figure S9e-h are shown in Figure S9i-l. The experimental results validate that Q4GOM is capable of fast live cell imaging and can serve as a powerful tool for visualizing and evaluating even low changes in the cell dry mass occurring in some dynamic cell processes. The measurement was performed with the time step of 1 second appropriate for the realized experiment, but the imaging speed can be significantly increased and it is limited only by the frame rate of the detector.

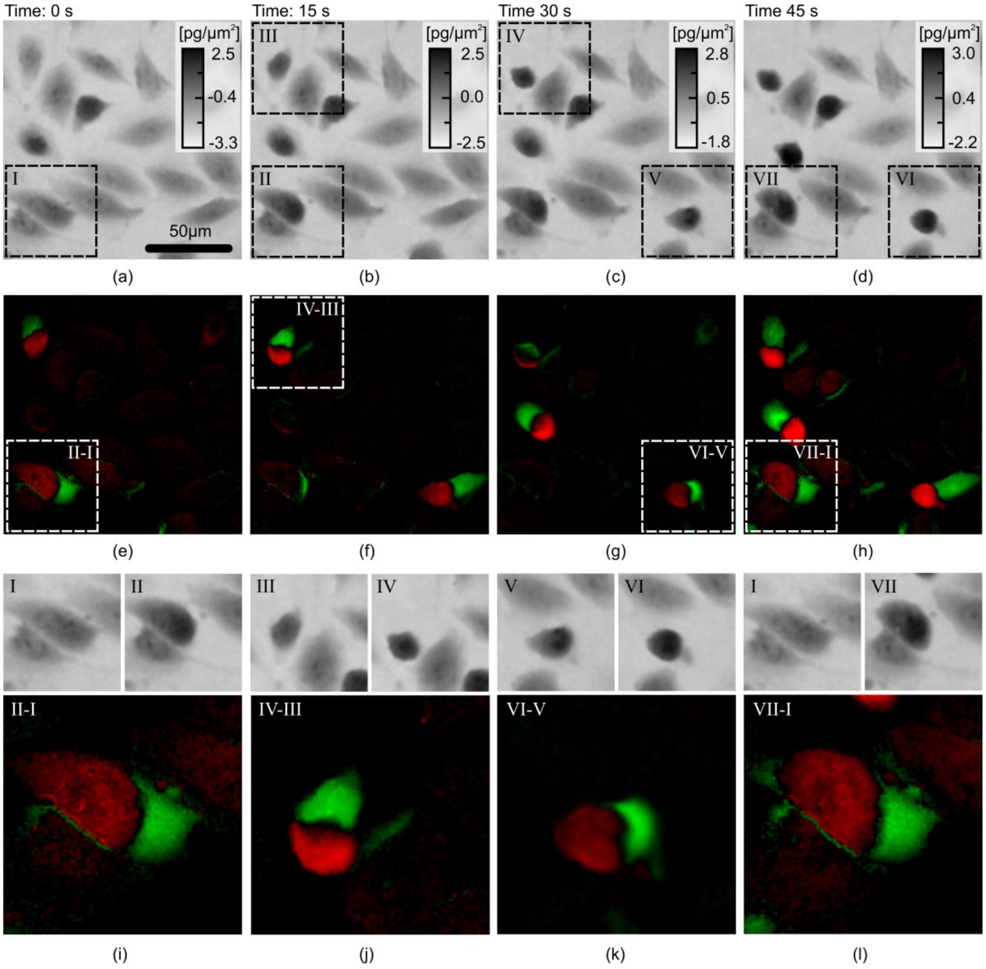

Figure S9 Morphological changes of LW3K12 cells induced by trypsin and their visualization using dynamic phase differences. (a)-(d) Quantitative phase images of LW3K12 cells separated by time step of 15 seconds. (e)-(g) Visualization of the differences in the dry mass density between related neighboring images (a)-(d). (h) Visualization of cumulative differences in the cell dry mass density between initial state (a) and final state (d). (i)-(l) Enlarged images of areas marked in (a)-(h). The red and green colors indicate areas where the dry mass density has increased and decreased, respectively. Images (a)-(d) are shown in units of pg/μm^2 recalculated from phase in radians according to Davies [4].

References

1 F. Gori,“Measuring Stokes parameters by means of a polarization grating,” Opt. Lett. **24**(9), 584–586 (1999).

2 J. Tervo, and J. Turunen,“Paraxial-domain diffractive elements with 100% efficiency based on polarization gratings,” Opt. Lett. **25**(11), 785–786 (2000).

3 T. Zikmund, L. Kvasnica, M. Týč, A. Křížová, J. Čolláková, and R. Chmelík,“Sequential processing of quantitative phase images for the study of cell behaviour in real-time digital holographic microscopy,” Journal of Microscopy **256**(2), 117–125 (2014).

4 H. G. Davies, and M. H. F. Wilkins,“Interference microscopy and mass determination,” Nature **169**, 541 (1952).

5 A. Křížová, J. Čolláková, Z. Dostál, L. Kvasnica, H. Uhlířová, T. Zikmund, P. Veselý, and R. Chmelík,“Dynamic phase differences based on quantitative phase imaging for the objective evaluation of cell behavior,” Journal of Biomedical Optics **20**(11), 111214 (2015).
